# Proteomics and Metabolomics in Varicocele-Associated Male Infertility: Advancing Precision Diagnostics and Therapy

**DOI:** 10.3390/jcm13237390

**Published:** 2024-12-04

**Authors:** Aris Kaltsas, Athanasios Zikopoulos, Eleftheria Markou, Athanasios Zachariou, Marios Stavropoulos, Zisis Kratiras, Evangelos N. Symeonidis, Fotios Dimitriadis, Nikolaos Sofikitis, Michael Chrisofos

**Affiliations:** 1 Third Department of Urology, Attikon University Hospital, School of Medicine, National and Kapodistrian University of Athens, 12462 Athens, Greece; ares-kaltsas@hotmail.com (A.K.); stamarios@yahoo.gr (M.S.); zkratiras@gmail.com (Z.K.); 2 Department of Obstetrics and Gynecology, Royal Cornwall Hospital, Truro TR1 3LJ, UK; athanasios.zikopoulos1@nhs.net; 3 Department of Microbiology, University Hospital of Ioannina, 45500 Ioannina, Greece; eleftheria.markou4@gmail.com; 4 Laboratory of Spermatology, Department of Urology, Faculty of Medicine, School of Health Sciences, University of Ioannina, 45110 Ioannina, Greece; azachariou@uoi.gr (A.Z.); nsofikit@uoi.gr (N.S.); 5 Department of Urology II, European Interbalkan Medical Center, 55535 Thessaloniki, Greece; evansimeonidis@gmail.com; 6 Department of Urology, Faculty of Medicine, School of Health Sciences, Aristotle University of Thessaloniki, 54124 Thessaloniki, Greece; helabio@yahoo.gr

**Keywords:** varicocele, male infertility, proteomics, metabolomics, biomarkers, seminal plasma, spermatozoa, oxidative stress, personalized medicine, diagnostic tools

## Abstract

**Background/Objectives:** Varicoceles are a common contributor to male infertility, significantly impacting male-factor infertility cases. Traditional diagnostic methods often lack the sensitivity to detect the molecular and cellular disruptions caused by varicoceles, limiting the development of effective, personalized treatments. This narrative review aims to explore the advancements in proteomics and metabolomics as innovative, non-invasive diagnostic tools for varicocele-associated male infertility and their potential in guiding personalized therapeutic strategies. **Methods:** A comprehensive literature search was conducted using databases such as PubMed, Scopus, and Web of Science up to October 2024. Studies focusing on the application of proteomic and metabolomic analyses in varicocele-associated male infertility were selected. The findings were critically analyzed to synthesize current knowledge and identify future research directions. **Results:** Proteomic analyses revealed differentially expressed proteins in the sperm and seminal plasma of varicocele patients, revealing disruptions in pathways related to oxidative stress, mitochondrial dysfunction, apoptosis, and energy metabolism. Key proteins such as heat shock proteins, mitochondrial enzymes, and apoptotic regulators were notably altered. Metabolomic profiling uncovered specific metabolites in seminal plasma—such as decreased levels of lysine, valine, and fructose—that correlate with impaired sperm function and fertility potential. The integration of proteomic and metabolomic data provides a comprehensive molecular fingerprint of varicocele-induced infertility, facilitating the identification of novel biomarkers for early diagnosis and the development of personalized therapeutic interventions. **Conclusions:** Advances in proteomics and metabolomics have significantly enhanced our understanding of the molecular mechanisms underlying varicocele-associated male infertility. These “omics” technologies hold great promise for improving diagnostic accuracy and personalizing treatment, ultimately leading to better outcomes for affected men. Future large-scale clinical trials and validations are essential to confirm these biomarkers and facilitate their integration into routine clinical practice.

## 1. Introduction

**Figure 1:**
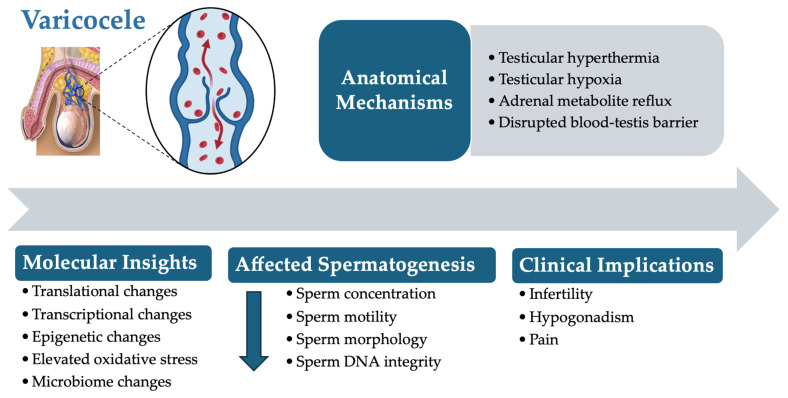
Anatomical, molecular, and diagnostic features associated with varicoceles.

Varicoceles, characterized by the abnormal enlargement of the pampiniform plexus veins within the spermatic cord, are among the most prevalent causes of male infertility [1]. Affecting approximately 15% of men globally and contributing to up to 40% of male-factor infertility cases, varicocele formation disrupts the delicate testicular environment [2,3]. Physiological challenges such as elevated scrotal temperatures, increased oxidative stress, and hormonal imbalances collectively impair spermatogenesis [4,5]. Consequently, key sperm parameters including concentration, motility, and morphology are adversely affected, significantly diminishing male fertility potential [6,7]. As illustrated in Figure 1, a varicocele involves complex anatomical abnormalities and molecular disruptions that complicate diagnosis and treatment.

Current clinical diagnostic methods, such as semen analysis and DNA fragmentation tests, provide general insights into sperm health by assessing parameters like sperm count, motility, and morphology. However, these methods lack the sensitivity to detect the subcellular and molecular disruptions induced by varicoceles [8]. While advanced tests for reactive oxygen species (ROS) levels, total antioxidant capacity, and DNA fragmentation offer additional information on oxidative stress and genomic integrity, they still do not capture the full spectrum of biochemical and molecular changes induced by varicoceles [9,10]. Important molecular alterations—such as mitochondrial dysfunction, apoptosis, and post-translational modifications—often remain undetected [11].

To address these limitations, the integration of “omics” technologies—specifically proteomics and metabolomics—offers a promising solution. Proteomics involves the large-scale analysis of proteins, which are crucial for executing cellular functions, while metabolomics examines small molecules or metabolites that represent the final products of cellular processes. By mapping molecular changes at the protein and metabolite levels, these approaches provide a molecular-level perspective on cellular health and function [12,13]. In the context of varicoceles, proteomics can reveal changes in protein expression and post-translational modifications in sperm and seminal plasma, whereas metabolomics offers real-time insights into the physiological state of the reproductive system by analyzing metabolites in seminal plasma. Together, these methods enable the identification of novel biomarkers for more sensitive and personalized diagnostic and therapeutic strategies [14].

Despite some success with treatments like varicocelectomy, outcomes remain inconsistent due to the complex and poorly understood pathophysiology of varicoceles. Without comprehensive tools to detect and analyze these molecular disruptions, clinicians lack crucial insights into the extent of infertility associated with varicoceles, which hinders the development of personalized treatment plans. Consequently, this may contribute to the inconsistent outcomes observed with surgical interventions [15].

This diagnostic gap presents a significant challenge in varicocele management. This manuscript will explore the potential of proteomics and metabolomics in advancing male infertility diagnostics and treatment. By moving beyond traditional semen analysis parameters, these “omics” methods promise the early detection and precise monitoring of varicoceles’ impact on fertility. Ultimately, the integration of proteomics and metabolomics could improve management strategies and enable personalized treatment, enhancing outcomes for men affected by this common condition.

## 2. Materials and Methods

A comprehensive literature search was conducted to gather relevant studies on the application of proteomics and metabolomics in varicocele-associated male infertility. The databases searched included PubMed, Scopus, and Web of Science, selected for their extensive biomedical and scientific coverage and their relevance to studies in reproductive health, proteomics, and metabolomics. These databases were chosen due to their accessibility and the breadth of indexed journals pertinent to our research focus. The literature search spanned up to October 2024. While Embase and the Cochrane Library are important resources, they were not included in our strategy. This exclusion was based on the specific focus of our study and the determination that PubMed, Scopus, and Web of Science would sufficiently capture the relevant literature on proteomics and metabolomics related to varicocele-associated male infertility. Embase primarily covers pharmacological and biomedical literature, and the Cochrane Library specializes in systematic reviews and clinical trials, which were outside the scope of our narrative review. Additionally, only articles published in English were considered, potentially omitting relevant research in other languages.

Keywords and Search Strategy: The search was conducted using keywords such as “varicocele”, “male infertility”, “proteomics”, “metabolomics”, “biomarkers”, “seminal plasma”, “spermatozoa”, “oxidative stress”, and “personalized medicine”. These terms were combined using Boolean operators (AND, OR) to enhance the precision of results. Additionally, Medical Subject Headings (MeSH) terms were employed in PubMed to ensure comprehensive retrieval of relevant articles.

The titles and abstracts of the identified studies were independently evaluated by two reviewers (A.K. and Athanasios Zikopoulos) to determine their eligibility based on the inclusion criteria. Studies not meeting the criteria were excluded at this stage. Full-text versions of potentially eligible articles were subsequently retrieved for a detailed assessment. Discrepancies in study selection were addressed through consensus discussions between the two reviewers. If disagreements persisted, a third reviewer (E.M.) was consulted to ensure methodological accuracy and minimize selection bias. The final list of studies was critically reviewed by all authors to confirm alignment with the research objectives.

Inclusion and Exclusion Criteria: The selection criteria focused on original research articles, reviews, and clinical studies relevant to varicocele-associated male infertility and the use of proteomic and metabolomic analyses. Specific inclusion criteria were as follows:

- Types of Studies: Both clinical and experimental studies were included to encompass a range of research methodologies. Clinical studies provided direct insights into biomarkers and potential diagnostic applications, while experimental studies contributed mechanistic insights into molecular alterations.
- Study Focus: Studies must have addressed either proteomic or metabolomic analyses in relation to varicocele-associated infertility, with a focus on biomarkers, molecular mechanisms, or potential diagnostic or therapeutic applications.
- Population: Studies involving male patients with varicocele or varicocele-associated infertility.
- Publication Date and Language: Studies published in English up to October 2024 were included. Articles with inadequate relevance, low methodological rigor, or insufficiently detailed molecular data were excluded.

**Figure 2:**
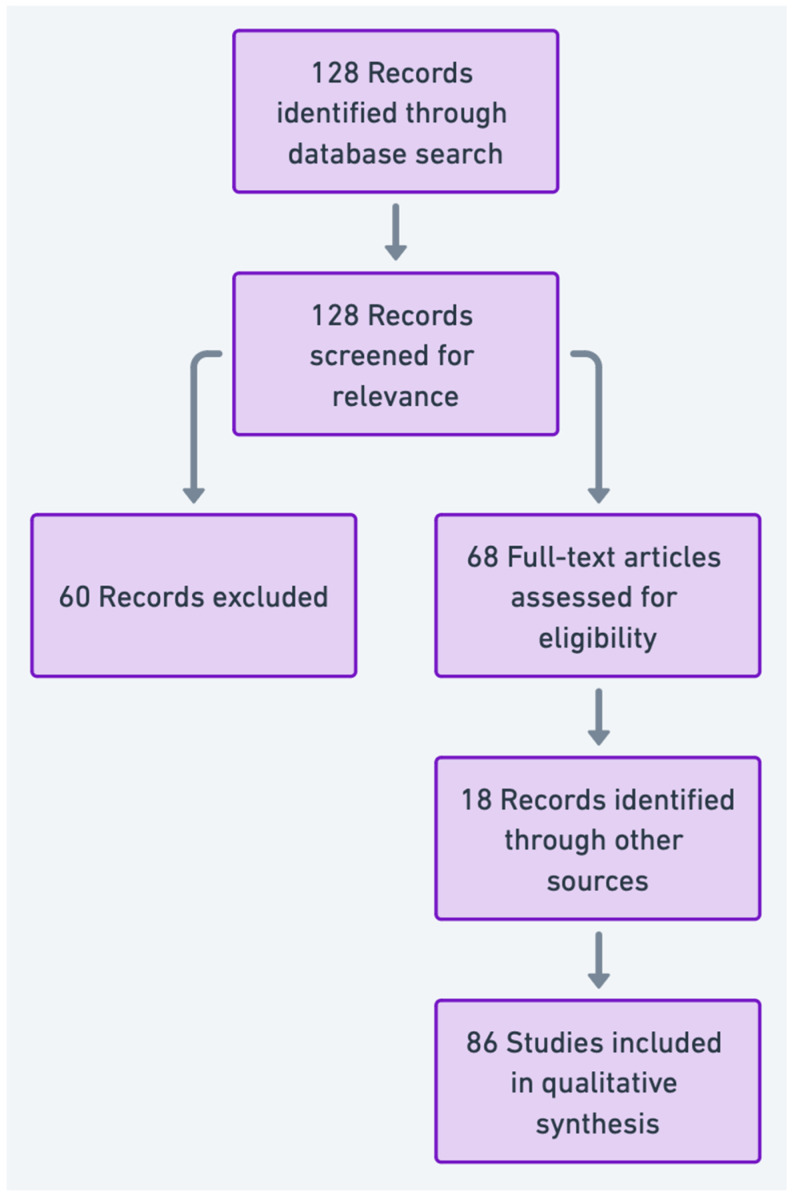
Prisma flow diagram illustrating the article selection process for studies on proteomics and metabolomics in varicocele-associated male infertility.

Data Extraction and Analysis: Relevant findings were extracted from selected articles, including specific biomarkers, affected molecular pathways, and implications for diagnosis and treatment. The findings were synthesized to identify trends, gaps, and future directions in the application of “omics” technologies for varicocele-associated male infertility. The PRISMA flowchart summarizing the article selection process is presented in Figure 2.

## 3. Proteomic Advances in Understanding Varicocele-Associated Male Infertility

Proteomics has emerged as a powerful tool in elucidating the molecular mechanisms underlying male infertility, particularly in conditions like varicoceles. Semen samples are ideal for proteomic studies due to the reliance of spermatozoa on proteins to execute essential physiological functions, given their lack of transcriptional activity. These proteins are involved in various molecular processes, including energy metabolism, post-translational modifications, DNA repair, and responses to oxidative stress [16,17,18]. Seminal plasma proteins also play a critical role in maintaining sperm function, as they are primarily derived from the testes and accessory sex glands, resulting in a high protein concentration (35–55 g/L), with about 80% being semenogelins [19,20,21]. Alterations in the expression of these proteins can directly affect sperm health and homeostasis.

Despite the potential of proteomics, several limitations should be acknowledged up front. A major challenge is the detection and quantification of low-abundance proteins that may have significant diagnostic and therapeutic value but are often masked by the high abundance of other proteins in seminal plasma. Current proteomic techniques, such as mass spectrometry and two-dimensional electrophoresis, often require enrichment or fractionation steps to overcome this limitation [22,23]. Advances in methodologies, including the use of targeted proteomics and enhanced bioinformatics tools, are improving sensitivity but remain constrained by sample complexity and variability [24].

### 3.1. Proteomics in Diagnosing Male Infertility

Proteomic analysis offers significant potential in identifying differential protein expression in semen, serving as a non-invasive biomarker tool for diagnosing male infertility associated with abnormal semen characteristics [25].

#### 3.1.1. Seminal Plasma Protein Markers in General Male Infertility

In azoospermic men, certain seminal plasma proteins are absent, such as acid phosphatase prostate (ACPP), prostate-specific antigen (KLK3), clusterin (CLU), zinc-alpha-2-glycoprotein (AZGP1), and glycodelin (PAEP) [26,27]. Drabovich et al. confirmed testis-expressed protein 101 (TEX101) as a biomarker for azoospermia and extracellular matrix protein 1 (ECM1) to differentiate non-obstructive azoospermia from vasectomy patients [28]. In cases of asthenozoospermia, protein tyrosine phosphatase non-receptor type 14 (PTPN14) shows dysregulation, while cystatin-C (CST3) levels are reduced [29,30]. In contrast, patients with oligoasthenozoospermia exhibit elevated levels of KLK3 and semenogelin-1 (SEMG1) [30]. Key proteins associated with sperm function, such as NPC intracellular cholesterol transporter 2 (NPC2), galectin-3-binding protein (LGALS3BP), lipocalin-1 (LCN1), and prolactin-inducible protein (PIP), show reduced expression in oligoasthenoteratozoospermia (OAT) [31].

#### 3.1.2. Proteomic Insights into Oxidative Stress and DNA Damage

Oxidative stress, a prominent factor in varicocele-associated male infertility, is commonly assessed in clinical settings through markers such as ROS levels, total antioxidant capacity (TAC), and malondialdehyde (MDA) concentrations in semen [32]. These markers help evaluate the extent of oxidative damage but do not provide detailed molecular insights into the affected proteins or pathways that underlie the cellular impairments seen in infertility [33].

Proteomic studies add value by identifying specific differentially expressed proteins (DEPs) involved in stress responses and regulatory pathways in men with elevated seminal ROS levels [30]. For example, Sharma et al. observed that membrane metallo-endopeptidase (MME) was present in the seminal plasma of ROS-positive men but absent in ROS-negative individuals. Conversely, fibronectin 1 (FN1) and macrophage migration inhibitory factor (MIF) were found only in ROS-negative samples, suggesting a complex molecular response to oxidative stress in sperm and seminal plasma [30].

Proteomic insights also provide a deeper understanding of DNA damage beyond what is offered by DNA fragmentation assays alone. A bioinformatic analysis of proteomic data by Intasqui et al. identified several potential markers of sperm nuclear DNA damage, such as solute carrier family 2 member 14 (SLC2A14), phosphoglycerate kinase 2 (PGK2), outer dense fiber protein 1 (ODF1), clusterin (CLU), and voltage-dependent anion-selective channel proteins 2 and 3 (VDAC2 and VDAC3). These proteins are associated with pathways that regulate DNA repair, oxidative stress responses, and cellular stability, offering insights into the molecular mechanisms disrupted by varicocele formation [34,35].

By combining conventional oxidative stress markers with proteomic data, clinicians may better understand the molecular disruptions in varicocele patients, allowing for a more targeted and personalized approach to treatment.

### 3.2. Proteomic Analysis in Varicocele Patients

While research on sperm proteomics in varicocele patients has been limited, recent studies have provided valuable insights into the molecular alterations associated with this condition.

#### 3.2.1. Sperm Proteomic Alterations in Varicocele Patients

Early protein profiling studies compared normozoospermic men without varicoceles to oligozoospermic men with the condition using techniques like two-dimensional gel electrophoresis (2D-GE), a laboratory method that separates proteins based on their size and electrical charge [36]. These studies identified DEPs mainly related to mitochondrial function, cytoskeletal structure, and heat shock responses [36]. Subsequent research investigated changes in sperm protein expression in varicocele patients before and after varicocelectomy, revealing significant increases in mitochondrial ATP synthase subunit delta (ATP5D), antioxidant enzyme superoxide dismutase 1 (SOD1), and heat shock protein family A member 5 (HSPA5) following surgery [37].

Advanced proteomic approaches like liquid chromatography–tandem mass spectrometry (LC-MS/MS), an advanced laboratory technique used to identify and measure proteins, have uncovered additional molecular mechanisms in varicocele formation [38]. Proteomic analyses have shown that a significant proportion of DEPs related to sperm function and energy metabolism are downregulated in both unilateral and bilateral varicocele cases [39]. Researchers identified 141 mitochondrial proteins in sperm, with 22 associated with mitochondrial structure and function being altered in varicocele patients [40]. Lower expression levels of proteins such as Na^+^/K^+^ ATPase alpha 4 (ATP1A4), heat shock-related 70 kDa protein 2 (HSPA2), sperm-associated antigen 17 (SPA17), and apolipoprotein A1 (APOA1) were correlated with reduced mitochondrial efficiency [40].

#### 3.2.2. Unilateral vs. Bilateral Varicocele Proteomic Profiles

##### Unilateral Varicoceles

Unilateral varicoceles predominantly affect the left side in approximately 90% of cases [41]. Comparative proteomic studies between fertile men and unilateral varicocele patients revealed 369 DEPs. Many of these proteins are involved in essential cellular processes such as ion binding, oxidoreductase activity, and pathways like small-molecule metabolism, stress response, and signal transduction [42]. Alterations in these proteins impact the physiological functions of sperm, including post-translational modification, free-radical scavenging, protein ubiquitination, and mitochondrial function. A set of 29 proteins linked to reproductive functions—such as maturation, motility, capacitation, and acrosome reaction—showed significant changes. Notable proteins include calcium-binding tyrosine phosphorylation-regulated protein (CABYR), A-kinase anchor proteins (AKAP), APOA1, semenogelin-1 (SEMG1), and sperm surface protein Sp17 (SPA17), which have been highlighted as potential biomarkers for unilateral varicocele [43].

##### Bilateral Varicoceles

In bilateral varicocele patients, unique proteomic profiles distinguish them from fertile individuals. The absence of APOA1, reduced expression of translocase of outer mitochondrial membrane 22 (TOM22), and increased expression of transglutaminase-4 (TGM4) correlate with oxidative stress and DNA fragmentation [44]. Proteins related to reproductive functionality, including outer dense fiber protein 2 (ODF2), tektin-3 (TEKT3), t-complex protein 11 homolog (TCP11), and calmegin (CLGN), display altered expression patterns, impairing the sperm’s fertilization capacity. Additionally, proteins such as enkurin (ENKUR), semenogelin-1 and -2 (SEMG1/2), sperm adhesion molecule 1 (SPAM1), and CABYR serve as indicators of reduced semen quality in bilateral varicocele patients [42].

Comparative profiling has shown 253 DEPs between unilateral and bilateral varicocele cases. These proteins are involved in pathways related to metabolism, apoptosis, and signal transduction. The dysregulation of sperm functions such as capacitation, hyperactivation, zona pellucida binding, and fertilization-related activities is more pronounced in bilateral varicocele cases due to the differential expression of proteins like glutathione S-transferase Mu 3 (GSTM3), sperm protein associated with the nucleus on the X chromosome B/F (SPANXB/F), cytochrome b5 reductase 2 (CYB5R2), CLGN, and Parkinsonism-associated deglycase (PARK7/DJ-1) [42]. Over half of these DEPs are linked to acetylation, suggesting that the downregulation of proteasome complex proteins could lead to increased DNA damage [45]. Proteins associated with acetylation involved in fertilization and acrosome reaction (transaldolase 1 [TALDO1], histone cluster 1 H2B family member B [HIST1H2B], and glucosamine-6-phosphate deaminase 1 [GNPDA1]), apoptosis and DNA damage (heat shock protein HSP 90-beta [HSP90AB1], serine/threonine-protein phosphatase 5 [PPP5C], and RuvB-like 2 [RUVBL2]), and mitochondrial dysfunction (succinate dehydrogenase complex flavoprotein subunit A [SDHA], peroxiredoxin-1 [PRDX1], and glutathione reductase [GSR]) have been proposed as post-translational protein biomarkers in varicocele patients [45].

### 3.3. Seminal Plasma Proteomics in Varicocele Patients

The proteomic profile of seminal plasma is crucial for enhancing sperm’s fertilization capacity [19]. Seminal plasma provides a supportive environment for sperm maturation and reflects overall male reproductive health. Approximately 10% of seminal plasma proteins originate from the testes, offering insights into testicular function [23]. These proteins are involved in critical stages of fertilization, including hyperactivation, capacitation, the acrosome reaction, and sperm–egg interaction [46]. Seminal plasma also contains about 30% of the proteins found in sperm, serving as an indicator of sperm functionality [24].

#### 3.3.1. Proteomic Biomarkers in Adolescents and Adults

##### Adults with Varicoceles

The initial study on seminal plasma proteomics in adult varicocele patients using two-dimensional sodium dodecyl sulfate–polyacrylamide gel electrophoresis (2D SDS-PAGE) identified 95 proteins with altered expression. Among these, proteins involved in inflammatory response, proteolysis, apoptosis regulation, sperm maturation, and sperm-egg fusion showed notable dysregulation [22]. Pathways related to nitric oxide metabolism and tetratricopeptide repeat domain binding activity were significantly increased, highlighting the adverse effects of varicocele formation on semen quality and sperm functional integrity [47].

##### Adolescents with Varicoceles

The prevalence of varicoceles shows an age-related increase, affecting approximately 7.9% to 16.2% of adolescents between the ages of 10 and 19, with rates reaching up to 19% by the age of 19 [48,49]. In adolescents with varicoceles and compromised semen quality, seminal plasma proteins essential for normal sperm function exhibit differential expression. Proteins related to sperm motility and capacitation, such as semenogelin I (SEMG1) and prostate-specific antigen (PSA), are overexpressed and underexpressed, respectively [50]. Belardin et al. indicated that the proliferation–apoptosis balance was disrupted in adolescents with varicoceles [51]. Insulin-like growth factor-binding protein 7 (IGFBP7), associated with proliferation, was overexpressed, while deoxyribonuclease-1 (DNASE1), involved in apoptosis regulation, was underexpressed [51]. A high presence of immune response proteins in the seminal plasma indicated a chronic inflammatory state adversely impacting testicular function and semen quality [52].

#### 3.3.2. Proteomic Changes in Seminal Plasma Following Varicocelectomy

Changes in protein expression have been observed in the seminal plasma of patients after varicocelectomy. Post-surgical proteomic analysis identified 38 proteins with unique expression patterns. Pathways associated with oxidative stress response, gluconeogenesis, and protein stability were notably active following varicocelectomy. Key proteins such as PARK7/DJ-1, S100 calcium-binding protein A9 (S100-A9), superoxide dismutase 1 (SOD1), annexin A1 (ANXA1), glyceraldehyde-3-phosphate dehydrogenase (GAPDH), and malate dehydrogenase (MDH) were overexpressed, suggesting they play a role in re-establishing cellular balance after surgery [47]. Lower levels of negative elongation factor E (NELFE) indicated reduced oxidative stress, while the higher expression of transglutaminase-4 (TGM4) hinted at preserved sperm-binding function after varicocelectomy [50].

Further studies validated certain seminal plasma proteins as potential non-invasive biomarkers for diagnosing varicocele-associated infertility. In varicocele patients, the apoptosis-related protein B-cell lymphoma 2 (BCL2) was downregulated, while BCL2-associated X protein (BAX) was upregulated. An inverse relationship was found between BAX levels and sperm concentration, motility, and normal morphology, underscoring its relevance as a marker of compromised semen quality [53].

Table 1 summarizes key proteins identified in varicocele-associated male infertility, detailing their expression changes, biological functions, and potential as clinical biomarkers.

## 4. Metabolomics as Diagnostic Tool in Male Infertility

Metabolomics, the comprehensive analysis of small molecules known as metabolites within biological systems, has emerged as a valuable approach to understanding male infertility. Focusing on the non-targeted analysis of small molecules under 1 kDa—including hormones, signaling molecules, and secondary metabolites—metabolomics provides detailed insights into the physiological state of cells and tissues [54]. Unlike proteins and mRNA transcripts, metabolites are simpler in complexity yet represent the final products of cellular processes, exerting immediate influence on biological systems [55].

Both targeted and untargeted metabolomic approaches are employed to explore the metabolome. Targeted metabolomics quantifies specific sets of known metabolites, providing precise measurements of predefined compounds. Untargeted metabolomics aims to detect and quantify as many metabolites as possible without prior selection, offering a global view of the metabolic state. Numerous high-throughput platforms facilitate metabolomic profiling even with small sample volumes. Techniques such as nuclear magnetic resonance (NMR) spectroscopy—particularly ^1^H NMR—provide detailed structural information without destroying the sample. Mass spectrometry (MS) methods, including electrospray ionization mass spectrometry (ESI-MS) and direct-injection mass spectrometry (DI-MS), offer high sensitivity and specificity. Spectroscopic methods like Raman spectroscopy and near-infrared (NIR) spectroscopy allow for rapid, non-destructive analysis. To preserve sample integrity, biological samples are typically stored at −80 °C to prevent metabolic activity that could alter metabolite concentrations [56].

The metabolome reflects the genome’s functional output, making biofluids like seminal plasma ideal for metabolomic studies [57]. Seminal plasma is particularly valuable for assessing reproductive health, as it captures subcellular and molecular changes in sperm [58]. Metabolomic analysis can reveal metabolic signatures linked to semen quality and associated reproductive conditions. Changes in the metabolic composition of seminal plasma are closely related to the underlying causes of male infertility [59]. Comprehensive metabolite profiling in semen—from both sperm and seminal plasma—can detect shifts in molecular composition crucial for managing infertility [60]. Bioinformatics tools aid in mapping pathways connected to sperm pathophysiology, while chemometric analysis effectively distinguishes infertile men from fertile controls [61].

NMR spectroscopy of seminal plasma has identified lysine as a potential biomarker for diagnosing idiopathic male infertility [62]. Other metabolites such as valine, 2-hydroxyisovalerate, hippurate, and fructose are found to be reduced in men with idiopathic infertility [62]. Bonechi et al. utilized NMR spectroscopy combined with principal component analysis (PCA) to classify semen samples based on different infertility conditions, with leukocytospermic patients clustering together [63]. This approach demonstrates the potential of metabolomics in differentiating between various infertility etiologies.

In asthenozoospermia, characterized by reduced sperm motility, metabolomic profiling via Raman spectroscopy has demonstrated the ability to distinguish asthenozoospermic samples from normal samples with an 83% accuracy rate [64]. Elevated levels of metabolites such as 5α-cholesterol and 7-ketocholesterol are associated with oxidative stress in these patients, indicating potential targets for therapeutic intervention [58]. These findings have implications for clinical practices, as oxidative stress biomarkers identified through metabolomics can guide antioxidant therapies aimed at improving sperm motility and overall reproductive outcomes [65].

In cases of non-obstructive azoospermia, Gilany et al. employed gas chromatography–mass spectrometry (GC-MS) paired with advanced chemometric analysis to identify 36 distinctive chemicals in seminal plasma that differentiate between testicular sperm extraction (TESE)-positive and TESE-negative samples. Only five of these metabolites were cataloged in the Human Metabolome Database (version 3.6), including dimethyl-(1S)-bicyclo(3.1.1)hept-2-ene-2-methanol, 2-pyrrolidineacetic acid, and 4,5-dimethoxy-1,2-benzenedicarboxylic acid. These metabolites provide potential biomarkers for differentiating subtypes of azoospermia [66]. This differentiation not only aids in patient counseling but also minimizes unnecessary invasive procedures by identifying candidates more likely to benefit from TESE [67].

In patients with spinal cord injury (SCI), seminal plasma metabolomic analysis of lipids revealed molecules associated with nucleotide synthesis and hydrogen peroxide response pathways. These findings indicate that signal transduction is notably compromised in SCI patients, contributing to infertility [68]. The identification of these disrupted pathways highlights opportunities for targeted therapeutic interventions, such as enhancing nucleotide synthesis or mitigating oxidative stress to improve reproductive outcomes [69].

Overall, metabolomic findings are increasingly being integrated into clinical practices and therapeutic strategies. By combining metabolomic biomarkers with bioinformatics and traditional diagnostic parameters, clinicians can achieve more precise patient stratification and personalized interventions. For example, metabolomic profiles may guide antioxidant supplementation, ART optimization, or minimally invasive sperm retrieval approaches [67]. Such applications underscore the transformative potential of metabolomics in male infertility diagnosis and management.

Table 2 provides an overview of key metabolites associated with varicocele-induced infertility, detailing their roles in cellular function and potential as biomarkers.

## 5. Integration of Proteomics and Metabolomics for Diagnostic Innovation

Proteomics and metabolomics offer complementary perspectives for diagnosing varicocele-associated infertility by targeting different but interconnected molecular elements. Proteomics focuses on the profile and function of proteins in sperm and seminal plasma, revealing changes in protein expression that directly impact cellular function. Metabolomics examines small-molecule metabolites that provide insights into biochemical pathways disrupted by varicoceles, such as oxidative stress and energy metabolism [71]. For instance, a combined analysis of proteomic and metabolomic data identified oxidative stress as a key disruptor in varicocele formation, with proteomics highlighting the underexpression of antioxidant enzymes like superoxide dismutase (SOD1) and metabolomics detecting elevated levels of 7-ketocholesterol, a marker of oxidative damage. These findings have supported clinical decisions to incorporate antioxidant therapies tailored to specific biomarker profiles [58,72]. Integrating data from these two fields allows for a more comprehensive molecular fingerprint, enhancing diagnostic accuracy by identifying specific protein and metabolite biomarkers. This holistic approach paves the way for personalized treatment strategies, tailoring interventions based on each patient’s unique molecular profile [73,74].

Proteomic and metabolomic analyses in varicocele patients have identified several critical molecular pathways disrupted by the condition. One major affected pathway is oxidative stress, resulting from an excessive production of ROS that damages sperm DNA and cellular structures, leading to impaired fertility [75]. Additionally, mitochondrial dysfunction has emerged as a key factor, as proteins related to energy production are often underexpressed in varicocele patients, causing reduced ATP synthesis and affecting sperm motility and viability [40]. This interplay is evident in studies where proteomics identified reduced levels of mitochondrial proteins such as COX5B, while metabolomics confirmed decreased levels of citrate and lactate, critical intermediates of mitochondrial respiration and glycolysis, respectively. These findings have informed interventions targeting mitochondrial support to improve sperm viability [40,76]. Metabolomic studies complement this by identifying metabolites such as lactate, which reflects impaired glycolysis, and citrate, which is crucial for mitochondrial respiration. The reduction in these metabolites aligns with proteomic findings of decreased mitochondrial enzyme expression, providing a deeper understanding of how energy metabolism is compromised [40].

Bioinformatic analysis further reveals disruptions in signal transduction pathways and apoptosis, with altered protein expression impacting cellular communication and leading to premature cell death in sperm cells. One study combined proteomics and metabolomics to identify the differential expression of apoptotic regulators, such as Bax and Bcl-2, along with elevated levels of glycerylphosphorylcholine, a metabolite associated with membrane stability and sperm viability. This dual insight has guided personalized therapeutic strategies to mitigate apoptosis and improve membrane integrity in varicocele patients [70,75]. Collectively, these insights provide a framework for understanding the molecular basis of varicoceles’ impacts on male infertility.

As proteomics and metabolomics techniques continue to advance, their application to personalized fertility treatments is expected to increase. Molecular profiling could allow clinicians to classify varicocele patients based on specific proteomic and metabolomic markers, enabling more tailored interventions. For example, antioxidant therapies might be optimized for patients showing high oxidative stress biomarkers, while others with significant mitochondrial dysfunction could receive treatments aimed at enhancing energy metabolism [70]. A notable example involves the integration of proteomic and metabolomic data to identify concurrent elevations of oxidative stress proteins (e.g., PRDX2) and metabolites (e.g., 7-ketocholesterol). Patients with these profiles have demonstrated better outcomes with combination therapies targeting both redox balance and mitochondrial function [9].

Ultimately, the integration of these “omics” technologies may facilitate the development of non-invasive diagnostic tools and personalized treatment plans, leading to improved outcomes for patients with varicocele-related infertility. The clinical validation of specific biomarkers will be crucial to translating these molecular insights into standard care, offering infertile men targeted treatments that enhance fertility based on their unique biomolecular profiles [73].

## 6. Challenges, Limitations, and Future Prospects of ‘Omics’ Technologies

The application of proteomics and metabolomics to male infertility, particularly varicocele-associated infertility, presents several technical and methodological challenges. A primary obstacle is the complexity of proteins in seminal samples, where high heterogeneity complicates detection and quantification, especially for low-abundance proteins that hold diagnostic value. Current proteomic techniques, like mass spectrometry (MS) and two-dimensional electrophoresis (2DE), require refinement to improve sensitivity for detecting these subtle biomarkers [77]. Advances in proteomic methodologies, including protein enrichment and customized techniques, now allow for the identification of low-abundance proteins and post-translational modifications such as glycosylation, phosphorylation, acetylation, and methylation in sperm and seminal plasma.

Metabolomics also presents challenges in terms of metabolite quantification due to the rapid turnover of small molecules and their sensitivity to environmental factors. High-throughput metabolomic analyses such as NMR and liquid chromatography–mass spectrometry (LC-MS) require advanced analytical methods to accurately distinguish metabolomic profiles. Recent advancements in bioinformatics and data processing have started addressing these issues, allowing for more precise interpretations of complex datasets and enhancing the reproducibility of results [67].

For the successful clinical translation of “omics” technologies, several critical steps must be undertaken. Biomarkers identified through proteomic and metabolomic studies require rigorous validation in large, diverse populations to ensure their reproducibility and reliability across demographic groups. Validation studies should focus on key markers, such as oxidative stress indicators (e.g., 7-ketocholesterol) and mitochondrial dysfunction markers (e.g., citrate), to confirm their diagnostic and prognostic utility in male infertility. Multicenter clinical trials are essential to substantiate these findings and demonstrate their relevance in clinical settings [78].

Regulatory approval processes represent another pivotal challenge in the integration of omics technologies into clinical practice. Omics-based diagnostic tests must comply with stringent regulatory standards, ensuring their safety, accuracy, and clinical relevance. The development of standardized protocols for sample collection, processing, and analysis will be critical to overcoming inter-study variability and facilitating the approval of these diagnostic tools by regulatory bodies such as the Food and Drug Administration (FDA) and the European Medicines Agency (EMA). Adherence to international guidelines for biomarker validation and assay standardization will further streamline the translation of these technologies into widespread clinical use [79].

Cost considerations also play a crucial role in the clinical adoption of omics technologies. The high costs associated with these advanced methodologies currently limit their accessibility in routine diagnostic settings. To address this, ongoing efforts aim to automate workflows and optimize high-throughput assays, which will reduce costs and improve scalability. Integrating omics-based biomarker testing into existing diagnostic frameworks, such as standard semen analyses, represents a practical and economically viable approach to ensuring accessibility for a broader range of patients [80].

Non-invasive diagnostic tools, such as exosome-based panels, represent a promising frontier in translating omics biomarkers into clinical practice. Exosomes, which carry proteins and metabolites reflective of their cell of origin, provide unique insights into sperm health and function. Emerging evidence suggests that exosomal proteins, such as HSP70 and TGF-β, can serve as biomarkers for oxidative stress and inflammation in varicocele-associated infertility. These exosome-based diagnostics could complement traditional semen analysis by offering more granular molecular insights and improving early detection and treatment monitoring [81].

Future developments in omics technologies hold significant promise for advancing the diagnosis and treatment of male infertility. Emerging tools, such as single-cell omics and artificial intelligence (AI)-based algorithms, are expected to revolutionize the field by enabling cell-specific analyses and improving the interpretation of complex datasets. Single-cell omics could provide unprecedented insights into spermatogenesis at the molecular level, while AI-powered models may enhance the predictive accuracy of diagnostic algorithms [82]. Furthermore, integrating microbiome research with proteomics and metabolomics could uncover novel microbial biomarkers that influence spermatogenesis and the integrity of the blood–testicular barrier, presenting new opportunities for therapeutic intervention [83,84,85].

By addressing these challenges systematically, including biomarker validation, regulatory compliance, cost-effectiveness, and the development of non-invasive diagnostics, omics technologies can transition from research to routine clinical practice. These advancements will enable personalized and precise diagnostic approaches, ultimately improving outcomes for patients with varicocele-associated infertility.

### Strengths and Limitations

This review presents a comprehensive examination of advancements in proteomics and metabolomics as diagnostic tools for varicocele-associated male infertility. A significant strength of this review is the integration of advanced “omics” technologies, offering a cutting-edge approach to identifying biomarkers associated with varicocele-induced infertility. These methods surpass conventional semen analysis by providing molecular-level insights that enhance the understanding of underlying pathophysiological mechanisms [70,81].

Another strength lies in the systematic and thorough literature search conducted using robust databases such as PubMed, Scopus, and Web of Science. This approach ensured the inclusion of relevant and high-quality studies, contributing to the reliability of the findings [70]. The emphasis on biomarkers highlights the diagnostic potential of proteomic and metabolomic analyses, paving the way for future advancements in personalized medicine and targeted therapeutic interventions [81].

However, several limitations should be acknowledged. First, the exclusion of non-English studies and certain databases (e.g., Embase and Cochrane) may have led to the omission of potentially relevant research, introducing language bias and limiting the comprehensiveness and generalizability of the findings. This constraint may affect the overall conclusions drawn from the review.

Second, there is significant heterogeneity among the included studies. Variability in methodologies, sample populations, and analytical techniques across different studies may impact the comparability of findings and the reproducibility of identified biomarkers [67,77]. This heterogeneity poses challenges in synthesizing results and drawing definitive conclusions.

Third, the translational application of the identified biomarkers faces challenges. Despite promising findings, the clinical implementation of proteomic and metabolomic biomarkers requires extensive validation and standardization, which remains a barrier to their adoption in routine clinical practice [86]. Large-scale, multicenter clinical trials are necessary to establish the reliability and predictive value of these biomarkers before they can be integrated into standard care.

Additionally, many studies included in this review focus on specific male infertility subtypes, which may not represent the broader population of individuals affected by varicocele-associated infertility [61,75]. This limitation in sample diversity may affect the generalizability of the findings to all varicocele patients.

Furthermore, as a narrative review, this study does not employ the systematic methodology required for a systematic review or meta-analysis. The inclusion of studies was based on relevance rather than a predefined protocol, potentially introducing selection bias. Reliance on published studies without access to raw data restricts the ability to perform quantitative analysis or independently verify the results, as findings are based on the interpretations and conclusions of the original authors.

## 7. Conclusions

Proteomics and metabolomics offer powerful, non-invasive approaches for diagnosing varicocele-associated infertility. These technologies provide a molecular-level view of cellular processes, enabling the identification of specific protein and metabolite biomarkers associated with oxidative stress, mitochondrial dysfunction, and impaired sperm function. By profiling these biomarkers in sperm and seminal plasma, researchers can elucidate the underlying mechanisms of infertility, offering diagnostic precision beyond traditional semen analysis.

The integration of “omics” approaches into clinical practice has the potential to revolutionize male infertility diagnostics by refining and expanding the parameters that can be assessed. These technologies support a shift toward personalized diagnostics and treatments, tailoring interventions based on each patient’s unique molecular profile. Individualized insights can guide clinical decisions, predict responses to treatments like varicocelectomy, and enable the development of targeted therapies that address the specific biological disruptions caused by varicoceles.

Looking ahead, the continued integration of proteomics and metabolomics in male infertility research is poised to deepen our understanding of complex infertility mechanisms and pave the way for novel, non-invasive diagnostic tools. As technology advances, combining proteomics, metabolomics, and emerging fields like exosomal proteomics will allow for the comprehensive profiling of male reproductive health. This convergence of “omics” technologies holds great promise for the future of male infertility diagnostics and treatment, potentially leading to highly effective, individualized care for patients affected by varicocele-associated infertility.

## Figures and Tables

**Table 1:** Key proteins identified in varicocele-associated male infertility.

Proteins	Expression Change	Biological Function	Clinical Implication/ Potential as a Biomarker	References
HSPA2	↓	Involved in sperm maturation and capacitation	Marker for impaired sperm function	[40,42,44]
ATP1A4	↓	Sperm motility and ion transport	Indicates reduced mitochondrial efficiency	[40]
APOA1	Ø/↓	Cholesterol transport and lipid metabolism	Marker for oxidative stress and mitochondrial dysfunction	[40,44]
CABYR	↓/↑	Sperm capacitation and motility	Potential biomarker for unilateral varicoceles	[42,44]
SEMG1	↑	Semen coagulation and sperm motility regulation	Indicator of altered semen quality	[42,50]
SEMG2	↓	Semen coagulation and sperm motility regulation	Indicator of altered semen quality	[42,50]
BAX	↑	Promotes apoptosis	Correlates with decreased sperm concentration and motility	[53]
BCL2	↓	Inhibits apoptosis	Reduced levels indicate increased apoptosis in sperm cells	[53]
PARK7/DJ-1	↑	Antioxidant properties, protects against oxidative stress	Marker for recovery after surgery	[45,47]
SOD1	↑	Antioxidant enzyme reducing oxidative stress	Indicates improved oxidative status after surgery	[37,47]

↑: Upregulated or increased expression/levels; ↓: downregulated or decreased expression/levels; Ø: absent (protein or metabolite not detected in the sample); ↑/↓: altered expression, indicates that expression levels can be either upregulated or downregulated depending on individual studies or patient variability; HSPA2: heat shock protein family A member 2; ATP1A4: ATPase Na^+^/K^+^ transporting subunit alpha 4; APOA1: apolipoprotein A-I; CABYR: calcium-binding tyrosine (Y) phosphorylation-regulated protein; SEMG1: semenogelin 1; SEMG2: semenogelin 2; BAX: BCL2-associated X protein; BCL2: B-cell lymphoma 2; PARK7/DJ-1: Parkinsonism-associated deglycase; SOD1: superoxide dismutase 1.

**Table 2:** Key metabolites identified in varicocele-associated male infertility.

Metabolites	Expression Change	Biological Function	Clinical Implication/ Potential as a Biomarker	References
Lysine	↓	Amino acid involved in protein synthesis	Potential biomarker for idiopathic infertility	[62]
Valine	↓	Amino acid involved in energy metabolism	Associated with impaired sperm function	[62]
Fructose	↓	Energy source for spermatozoa	Lower levels correlate with reduced sperm motility	[62]
5α-Cholesterol	↑ *	Steroid involved in membrane fluidity	Marker for oxidative stress in the sperm membrane	[58]
7-Ketocholesterol	↑ *	Oxidized cholesterol derivative	Indicates oxidative damage in sperm cells	[58]
Lactate	↓/↑	Energy metabolism intermediary	Reflects changes in glycolytic pathway activity	[70]
Glycerylphos-phorylcholine	↓/↑	Membrane lipid metabolism	Associated with membrane integrity and sperm viability	[70]

↑: Upregulated or increased expression/levels; ↓: downregulated or decreased expression/levels; ↑/↓: altered expression, indicates that expression levels can be either upregulated or downregulated, depending on individual studies or patient variability; *: in asthenozoospermia.
